# Ruptured heterotopic pregnancy in a natural conception cycle: a case report at the Yaounde central Hospital (Cameroon)

**DOI:** 10.11604/pamj.2013.16.106.3506

**Published:** 2013-11-18

**Authors:** Jeanne Hortence Fouedjio, Florent Ymele Fouelifack, Jovanny Tsuala Fouogue, Zacharie Sando

**Affiliations:** 1 Obstetrics and Gynecology Unit of the Yaounde Central Hospital-Cameroon; 2 Faculty of Medicine and Biomedical Sciences of the University of Yaounde 1- Cameroon; 3 Head of the pathology unit of the Yaounde Gyneco-Obstetric and pediatric hospital – Cameroon

**Keywords:** heterotopic, ectopic, pregnancy, hemoperitoneum, Cameroon

## Abstract

Heterotopic pregnancy is very rare under natural circumstances. We report the case of a 28 year old Gravida2 Para1001 woman at 9 weeks of pregnancy who consulted in emergency for acute pelvic pain following metrorrhagia. Physical exam revealed hemoperitoneum without shock. An emergency ultrasonography revealed two gestational sacs, one intra-uterine and the other extra-uterine. Laparotomy was done and the findings were: a ruptured right tubal pregnancy with 1,300 milliliters of hemoperitoneum, type B left utero-adnexal adhesions and an increased uterus consistent with a 9 weeks pregnancy. Right total salpingectomy was done and the patient did well postoperatively. That intrauterine pregnancy evolved normally under progesterone supply and the woman delivered a termed live female baby weighing 3.1 kilogrammes. In our context where ultrasound is not always available, practitioners carrying out salpingectomy for ruptured ectopic pregnancies should bear in mind the plausibleness of heterotopic pregnancy in order to properly handle the uterus.

## Introduction

Firstly reported in 1761 after an autopsy (Duvernay cited by Diallo and coll) [1], heterotopic pregnancy is the concomitant localization of intrauterine and extra-uterine pregnancies resulting from fecondation of two ovocytes expulsed at a short time interval and implantation of resulting blastocysts [1]. It is a very rare condition under natural circumstances with an incidence varying between 1 for 10,000 and 1 for 30,000 pregnancies [1–3]. The prevalence of heterotopic pregnancy in Cameroon is unknown. We report a case of continuation of an intrauterine pregnancy after surgical ablation of a concomitant tubal gestational sac at the Yaoundé Central Hospital.

## Patient and observation

**Figure 1:**
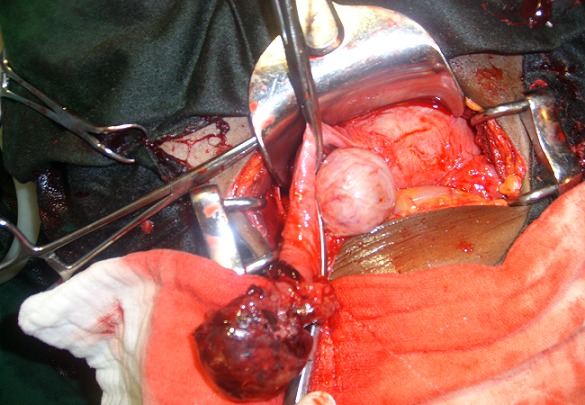
Operative findings after emergency laparotomy showing a ruptured left tubal pregnancy and an increased globular uterus

**Figure 2:**
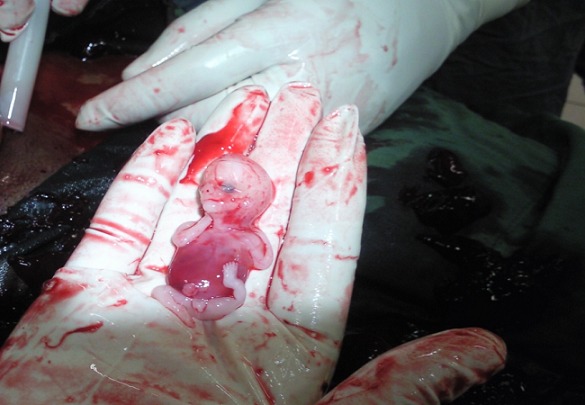
Operative findings after emergency laparotomy showing the extra-uterine embryo

Miss EJ, a 28 year old, single was received in our emergency unit for pelvic pain at 9 weeks and 5 days of pregnancy. The pain started five days prior to consultation and was associated with dark and light vaginal bleeding. She first consulted in a health center where she received antispasmodics without success. She was then referred to our emergency unit for better management. In past history, the patient had her first menses at 13 and the first sexual intercourse at 19. Her menstrual cycle is regular lasting 30 days and she bleeds for 5 days. She has no history of sexually transmitted infection. As contraception, she has always used male condom and practiced interrupted coïtus . Her first pregnancy resulted in a normal vaginal delivery at term. The ongoing pregnancy was the second and was at 9 weeks and 5 days. She is a dizygotic twin. She was successfully treated for pulmonary tuberculosis four years ago. On admission she had a good general condition and the vital parameters were normal. Gynecological examination revealed an enlarged uterus consistent with a 10 weeks pregnancy, a painful left adnexal mass of 10 centimeters of diameters and cervical motion tenderness. There was no vaginal bleeding. We suspected an ectopic pregnancy associated to uterine fibroids. Differential diagnoses were a complicated ovarian cyst in pregnancy and pelvic inflammatory disease in pregnancy. One hour after admission, while being in the imaging unit for emergency pelvic ultrasonography, she felt a sudden, intense and cramp-like pelvic pain associated with vomiting. The pain then diffused progressively to the whole abdomen. The findings of the ultrasonography were: an evolving singleton intrauterine pregnancy of 7 weeks and 6 days, a gestational sac in the right uterine adnexae containing a non living embryo of 8 weeks and 2 days and 300 milliliters of hemoperitoneum. On reevaluation, vital parameters remained normal. On abdominal palpation, we noted a slight pelvic tenderness. On vaginal digital exploration the cervix was long and closed, and adnexae were tender and difficult to palpate. The pouch of Douglas was painful and bulging. Culdocentesis brought back 4 milliliters of non clotting blood. We concluded that the extra-uterine pregnancy was ruptured. After an intramuscular injection of 500 milligrammes of long acting progesterone, we carried out an emergency laparotomy. We found a left tubal ruptured pregnancy, a homolateral corpus luteum, a globular and soft uterus consistent with a pregnancy of 9 weeks, 1,300 milliliters of hemoperitoneum and left adnexae embedded in type B adhesions (Figure 1, Figure 2). We did a total left salpingectomy. Postoperative course was uneventful and the patient was discharged five days after surgery. Every week she received an intramuscular dose of 500 milligrammes of long acting progesterone till the eighteenth gestational week. The course of the pregnancy was normal and our patient gave birth at term to a live girl weighing 3.1 kilogrammes.

## Discussion

There are two categories of risk factors for heterotopic pregnancy: risk factors of ectopic pregnancy (history of infertility, sexually transmitted infection, intrauterine device, smoking, hormonal contraception, pelvic surgery) and ovulation induction and assisted reproductive technologies (ART) [4]. Our patient did not have any risk factor of ectopic pregnancy. The prevalence of heterotopic pregnancies among those obtained after ART varies from 1 to 2.9% [5]. The frequency of twin deliveries among dizygotic twin women is the double of that in the general population and dizygotic pregnancy increases the risk of heterotopic localization [5]. Our patient conceived naturally but is predisposed to heterotopic pregnancy because she is dizygotic twin and has had a dizygotic pregnancy. Making early diagnosis of heterotopic pregnancy may not be easy in our context where first trimester and emergency room pelvic ultrasonography are not routinely performed. In those circumstances the most probable diagnosis will be that of a ruptured ectopic pregnancy leading to inappropriate handling of the uterus during surgery with adverse effects on the intrauterine foetus. Differential diagnosis include ruptured or twisted ovarian cyst in pregnancy and pelvic inflammatory disease in pregnancy [6]. Before the rupture, heterotopic pregnancy is diagnosed on the basis of amenorrhea, pregnancy related symptoms, pelvic pain (present in 82.7 to 90 per cent of cases), elevated level of β-human chorionic gonadotropin , signs of irritated peritoneum (present in 12.9 to 45 per cent of cases) and metrorrhagia (present in 50% of cases) [4, 7]. Pelvic ultrasonography confirms the diagnosis when an extra-uterine gestational sac is seen. It has been shown that transvaginal pelvic ultrasonography makes a correct diagnosis in 88.9% of cases. It misses the diagnosis when the ectopic pregnancy is not seen or wrongly seen as: an ovarian cyst, a pedonculated uterine fibroid undergoing necrobiosis, a hydrosalpinx, a twisted adnexae or an ovarian tumor [1]. Other misleading presentations of heterotopic pregnancy include threatened or incomplete abortion and voluntary termination of pregnancy [1]. Five days before the rupture of her heterotopic pregnancy our patient presented pelvic pain and light metrorrhagia. These could have been diagnosed as threatened abortion. After rupture, the diagnosis was evident for the team on call on the basis of evolution and the results of pelvic ultrasonography. In up to 50% of cases, heterotopic pregnancy can remain totally asymptomatic till it is discovered fortuitously by a routine first trimester ultrasound [8]. The treatment of heterotopic pregnancy can be surgical, medical or expectant. Out of 217 cases of heterotopic pregnancies reported in literature, 90.78% were managed surgically [1]. The procedure usually consists of salpingectomy via laparotomy or laparoscopy depending on patients’ hemodynamic state at the time of diagnosis. Depending on the state of the controlateral fallopian tube, on the patient's reproductive needs, and on the state of the ectopic pregnancy it is possible to consider conservation of the tube hosting the pregnancy. In our case, emergency laparotomy was carried out due to lack of equipment for laparoscopy and we did a total left salpingectomy because of important tubal damage. Heterotopic pregnancy can be managed medically under the following conditions: early and accurate diagnosis of the extra-uterine gestational sac and absence of symptoms [8]. Several modalities have been reported with success: ultrasound-guided vaginal aspiration or in situ injection of methotrexate, potassium chloride [9], or hyperosmolar glucose [10]. Expectant management of the ectopic pregnancy can be considered if it is not evolving [1].

Maternal outcome is appreciated through the following parameters: post-operative morbidity, complications of surgery and anesthesia, anemia, future fertility and death [1]. All these can be avoided by early diagnosis and medical or expectant management [8]. Our patient did well and was discharged on the sixth post operative day. Both ovaries and a macroscopically normal right fallopian tube were left in place with type B adhesions, making a mediocre prognosis for future fertility. Preserving mother's health and intra-uterine foetus were the goals of our management of this ruptured heterotopic pregnancy and we succeeded. Three main measures were implemented to preserve the intra-uterine pregnancy: shortest time under general anesthesia, proper handling of the uterus during surgery and supplementation with progesterone. A literature review found survival rate of 64.4% for the intra-uterine foetus. Miscarriage can occur and its frequency in the aforementioned series was 35.6% [1]. In our case the intrauterine foetus was born at term.

## Conclusion

In our context, emergency salpingectomy is usually done for ruptured ectopic pregnancy without first trimester pelvic ultrasonography. Considering the plausibleness of heterotopic pregnancy we recommend practitioners to carefully handle the uterus in this situation.
